# Impact of EMG Changes in Continuous Vagal Nerve Monitoring in High-Risk Endocrine Neck Surgery

**DOI:** 10.1007/s00268-015-3368-y

**Published:** 2015-12-17

**Authors:** Katrin Brauckhoff, Renate Vik, Lorentz Sandvik, John-Helge Heimdal, Turid Aas, Martin Biermann, Michael Brauckhoff

**Affiliations:** Department of Surgery, Haukeland University Hospital, Jonas Lies vei 65, 5021 Bergen, Norway; Department of ENT, Haukeland University Hospital, Bergen, Norway; Department of Clinical Medicine, University of Bergen, Bergen, Norway; Department of Radiology, Haukeland University Hospital, Bergen, Norway; Department of Clinical Science, University of Bergen, Bergen, Norway

## Abstract

**Background:**

Continuous vagal intraoperative neuromonitoring (CIONM) of the recurrent laryngeal nerve (RLN) may reduce the risk of RLN lesions during high-risk endocrine neck surgery such as operation for large goiter potentially requiring transsternal surgery, advanced thyroid cancer, and recurrence.

**Methods:**

Fifty-five consecutive patients (41 female, median age 61 years, 87 nerves at risk) underwent high-risk endocrine neck surgery. CIONM was performed using the commercially available NIM-Response 3.0 nerve monitoring system with automatic periodic stimulation (APS) and matching endotracheal tube electrodes (Medtronic Inc.). All CIONM events (decreased amplitude/increased latency) were recorded.

**Results:**

APS malfunction occurred on three sides (3 %). A total of 138 CIONM events were registered on 61 sides. Of 138, 47 (34 %) events were assessed as imminent (13 events) or potentially imminent (34 events) lesions, whereas 91 (66 %) were classified as artifacts. Loss of signal was observed in seven patients. Actions to restore the CIONM baseline were undertaken in 58/138 (42 %) events with a median 60 s required per action. Four RLN palsies (3 transient, 1 permanent) occurred: one in case of CIONM malfunction, two sudden without any significant previous CIONM event, and one without any CIONM event. The APS vagus electrode led to temporary damage to the vagus nerve in two patients.

**Conclusions:**

CIONM may prevent RLN palsies by timely recognition of imminent nerve lesions. In high-risk endocrine neck surgery, CIONM may, however, be limited in its utility by system malfunction, direct harm to the vagus nerve, and particularly, inability to indicate RLN lesions ahead in time.

## Introduction

The risk of recurrent laryngeal nerve (RLN) lesions during endocrine neck surgery depends on diagnosis, surgical procedure, the surgeon’s skills, and the management of the RLN itself [1, 2]. Even though intraoperative neuromonitoring (IONM) of the RLN is now routinely employed by many thyroid surgeons [2, 3], this has resulted in only a marginal reduction in RLN palsy rates [1–4]. The benefit of IONM seems to be greatest in more complex surgery such as reoperative surgery and thyroid cancer operations, but even under these circumstances, the number of patients needed to be treated to avoid a RLN lesion is relatively high [5].

One explanation for the limited effect of IONM on RLN lesion risk has been that IONM had relied on intermittent neurostimulation (INS). Most RLN lesions are caused by traction and pressure with a gradual deterioration of nerve function over time [6]. Most often, INS fails to detect a nerve lesion before it is complete [2]. In contrast, continuous vagal intraoperative neuromonitoring (CIONM) may reveal an imminent lesion timely enough to allow the surgeon to stop harmful dissection and allow the nerve to recover [7–9].

The potential of CIONM to effectively reduce risk to the RLN is based on a number of assumptions: (1) Nerve lesions develop over time so that the surgeon has a chance to react. (2) Imminent damage can be anticipated by specific changes in the electromyogram (EMG). (3) These changes in the EMG should be easy to distinguish from artifacts. Previous studies using either tube electrodes or translaryngeal needles to record the EMG signal from the intralaryngeal muscles have demonstrated that non-nerve damaging procedures may affect EMG parameters [6, 10, 11], but the clinical impact of such artifacts has not been examined yet.

Assuming that EMG changes are more frequent in complex surgery, we conducted a prospective study on CIONM during high-risk endocrine neck surgery characterizing and quantifying intraoperative laryngeal EMG changes and analyzing their impact on intraoperative surgical management and functional outcomes.

## Materials and methods

The study is part of a larger ongoing prospective study on IONM and voice changes during thyroid and parathyroid surgery. The study was approved by the Western Norwegian Regional Ethics Committee (REK Vest). All patients gave informed consent.

For the present analysis, we prospectively included patients undergoing the following procedures: (1) surgery for thyroid cancer with lymph node metastases in at least two cervical compartments [12], (2) surgery for large goiter with mediastinal extension potentially requiring sternotomy, and (3) reoperative surgery after previous ipsilateral thyroid or parathyroid surgery. The following were reasons for exclusion: (1) Preexisting lesion of the ipsilateral RLN, (2) tumor invasion of the RLN requiring nerve resection, and (3) withdrawal of informed consent.

All operations were performed under general anesthesia using propofol, sevoflurane, and fentanyl. Patients were intubated under 0.1 mg/kg intravenous vecuronium using a commercial EMG endotracheal tube (NIM FLEX™, Medtronic Xomed, Inc., Jacksonville, FL, USA) with an inner tube diameter between 7.0 and 8.0 mm. Translaryngeal needle electrodes were inserted when EMG tube was considered inappropriate (e.g., young age, substantial tracheal compression). The NIM-Response 3.0 system (Medtronic Inc., Minneapolis, MN, USA) was used both for CIONM and INS using impulses of 100 µs and 1 mA. For INS, a conventional handheld probe was used with four stimulations per second. For CIONM, the automated periodic stimulation (APS) system was employed with 10 stimulations per minute and impulses of 100 µs and 1 mA using 2- or 3-mm vagus electrodes.

**Fig. 1:**
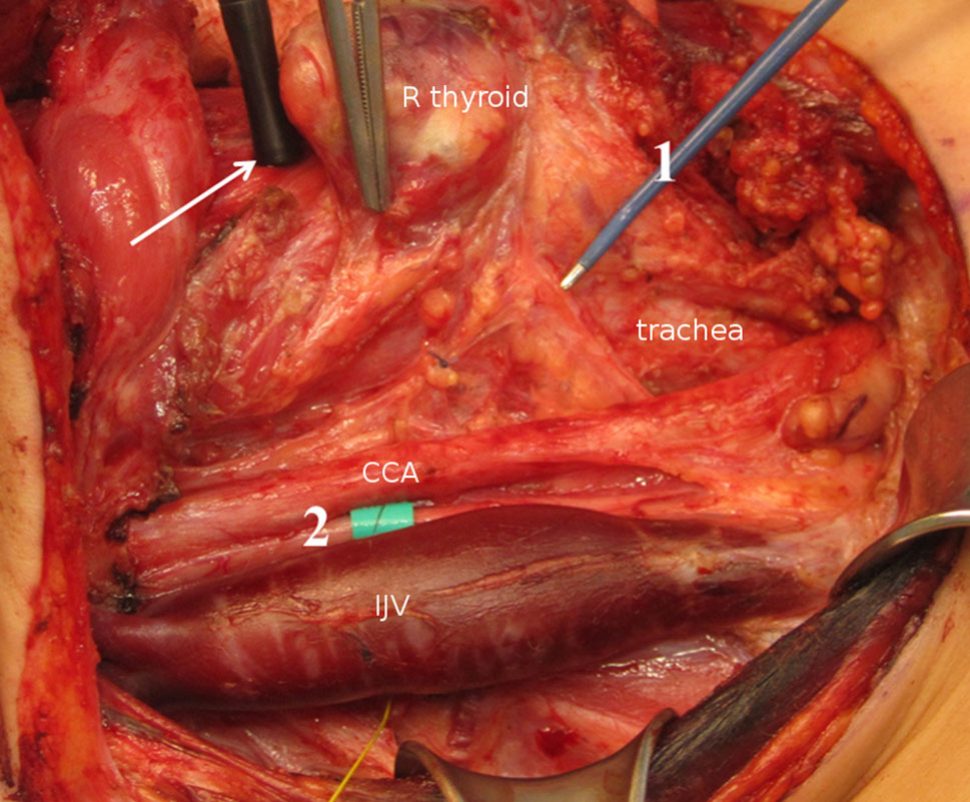
Intermittent neurostimulation (INS, 1) and continuous vagal intraoperative neuromonitoring (CIONM, 2) using a translaryngeal needle electrode (*white arrow*); CCA, common carotid artery; IJV, internal jugular vein. Thirteen-year-old female with MEN2B with bilateral medullary thyroid cancer and lymph node metastases in all cervical compartments

All patients underwent conventional surgery. Regardless of the approach medial or lateral to the large vessels, the vagus nerve was identified as early as possible and before any dissection near the RLN. The handheld stimulation probe (INS) was applied to the vagus nerve, and an EMG acquired from the endotracheal tube (V1). If the amplitude was below 300 µV, the position of the EMG tube was checked and corrected. The APS system was installed at this early point in surgery with the stimulation electrode positioned around the vagus nerve at the level of the crossing of the omohyoid muscle (Fig. 1). Baseline EMG amplitude and latency were determined as the mean values on 20 stimulations in accordance with the manufacturer’s specifications. Again, EMG amplitude was to exceed 300 µV. Alert value limits were left at their default values of amplitude reduction by more than 50 % and/or latency increase by more than 10 %. After establishing the APS system, the RLN was explored and stimulated (R1) before further dissection of the thyroid or parathyroid glands. Surgery started inferiorly except in large goiter. At the end of surgery, INS of the RLNs and the vagus nerves was performed and documented (R2, V2). The V1 and V2 stimulations were recorded at the highest accessible level of the vagus nerve, and proximally and distally to the APS electrode. Besides these fixed time points at start and end of surgery, INS could be used at any time in addition to CIONM if necessary. According to this dissection standard, thyroid dissection was classified into four localizations: L1 lower pole mobilization, L2 upper pole mobilization, L3 inferior dissection after luxation of the gland, and L4 dorsal dissection (ligament of Berry).

All CIONM events were recorded and classified: baseline amplitude reduction below 75, 50, 25 %, or loss of signal (LOS), as well as latency increase >10 %. Events with amplitudes <100 µV were counted as LOS regardless of the baseline amplitude and further subdivided into segmental (LOS type 1) or global (LOS type 2) [2]. For each event, we registered duration, presumed cause (e.g., traction, pressure, and thermic lesion), initiated action including time requirement, the result of the action, and the presumed relevance of the alert (intrinsic nerve lesion, artifact, or uncertain).

**Fig. 2:**
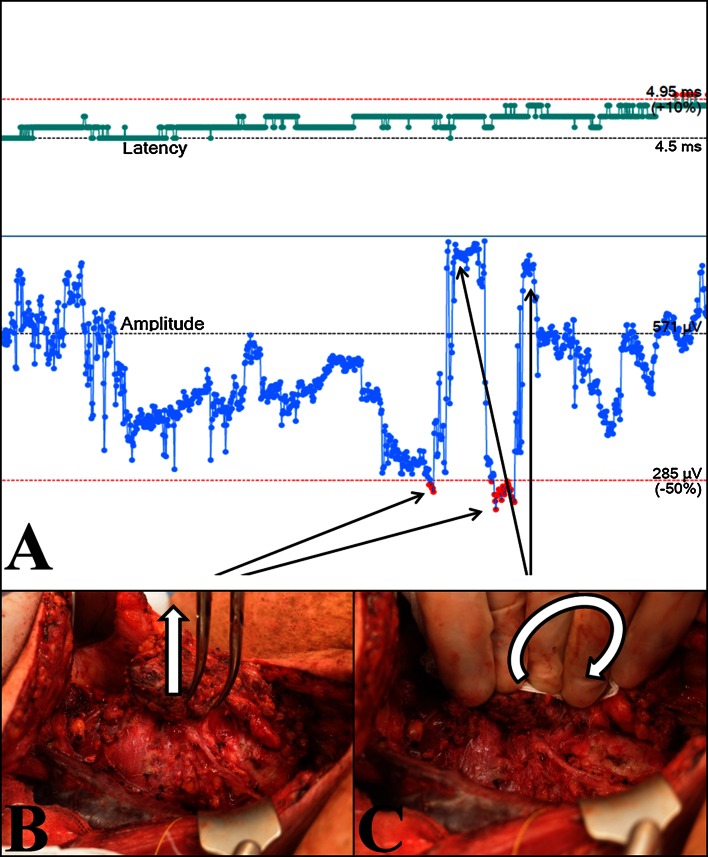
Effect of traction and pressure on the trachea on EMG amplitude using tube electrodes. Sixty-six-year-old female with papillary thyroid cancer. **a** EMG of the right RLN with baseline amplitude of 571 µV and latency of 4.5 ms. **b** Vertical traction on the thyroid lobe without traction of the RLN after complete mobilization in the ligament of Berry leads to an amplitude decrease of more than 50 % and triggers acoustic alert. **c** Manual luxation of the thyroid gland combined with pressure on the left side exerts the same traction on the trachea on the right side, but preserves the EMG signal with an even higher amplitude than at baseline

After signal alert, the surgical maneuver was stopped and all traction on the thyroid/tracheal complex discontinued immediately. To eliminate impaired contact between tube and laryngeal wall as a cause for artifacts (e.g., after partial decompression in large goiter), we first checked the effect of local pressure on cricoid and trachea on the signal parameters (Fig. 2). If this did not correct the problem, a complete system check was performed as recommended for LOS situations in conventional INS: stimulation with the hand probe, control of the APS electrode, check of laryngeal twitch, and/or control of the endotracheal tube [2, 13]. If this did not restore the signal, laryngeal needle electrodes were applied. If actual nerve damage was suspected, 300 mg hydrocortisone was administered intravenously. When electrodes were repositioned, new baseline values were only permitted if the new position provided at least 75 % of the initial baseline amplitudes.

All patients underwent flexible laryngoscopy before surgery and 1 day after by experienced ENT physicians blinded to the intraoperative events. Patients with affected vocal cord mobility were examined 3 and, if necessary, 6 months later. Persistent vocal cord palsy after 6 months was assumed to be permanent.

### Statistics

Data were managed and analyzed using SPSS for Windows (version 21 IBM Corporation, Armonk/NY). Distributions of continuous variables were expressed as medians and interquartile ranges. Between-group differences were tested for significance using the Chi-square test for categorical variables and Mann–Whitney–Wilcoxon rank-sum test for continuous variables, as appropriate. Statistical significance was set at *p* < 0.05 (two-sided).

## Results

Between November 2011 and November 2013, 56 consecutive patients meeting the inclusion criteria were operated using CIONM and enrolled in the study. One patient subsequently declined participation; consequently, the present analysis includes 55 patients (41 female and 14 male) with a median age of 61 years (interquartile range 46–70 years). Diagnoses and surgical treatment are summarized in Table 1. In total, there were 87 nerves at risk (NAR), 42 on the right and 45 on the left side.

**Table 1:** Demographics, diagnosis, surgery, and nerves at risk

Diagnosis	Patients (*n*)	Female (*n*)	Age (years)^a^	STX (*n*)	Redo (*n*)	Bilateral (*n*)	NAR (*n*)	Time (min)^a^
Thyroid cancer	26	20	56 (43;66)	3	5	26	47^b^	265 (185;345)
Mediastinal goiter	20	15	67 (52;75)	7	4	8	28	188 (130;214)
HPT	9	6	65 (52;67)	1	8	3	12	170 (145;195)
Total	55	41	61 (47;70)	11	17	37	87^b^	192 (165;262)

*HPT* hyperparathyroidism, *NAR* nerves at risk, *STX* sternotomy

^a^Median (interquartile range)

^b^Five nerves resected due to cancer invasion (three with preoperative palsy)

Laryngoscopy on the first postoperative day revealed three immobile vocal cords (3.4 % related to NAR) in three patients. Two were normal after 6 months, resulting in a permanent palsy rate of 1.1 % relative to NAR. One thyroid cancer patient required a transient tracheostomy. Postoperative bleeding occurred in two patients, a serious streptococcal wound infection occured in one, and permanent hypocalcemia occurred in one patient operated for thyroid cancer.

### Primary malfunction

APS could not be setup with either tube or needle electrodes on three of 87 sides (3.4 %) in three patients even though INS evoked normal signals. Two patients who underwent bilateral surgery showed normal CIONM signals on the other side and normal bilateral vocal cord mobility after surgery. The third patient suffered a true LOS confirmed by INS in the course of transsternal surgery for large goiter and had a transient RLN palsy postoperatively (Table 2).

**Table 2:** Clinical and electrophysiological data in patients with loss of signal

Patient [sex, age (years)]	Diagnosis	Surgical procedure	Loss of signal						V1 parameters		Postoperative vocal fold function
			Side	Type^a^	Locali-zation^b^	Cause	Evolution	Restitution during surgery	Amplitude [µV]	Latency (ms)	
Female, 77	Retrosternal MNG	HT right side	Right	1	L2	Traction	Acute	No	741	6.25	Transient palsy
Male, 66	PTC	TT + CND + LND	Right	1	L4	Thermic	Acute	No	461	4.5	Permanent palsy
Male, 51	PTC	TT + CND + LND	Right	2	L3/4	Traction	Gradual	No	327	5.13	Normal
Male, 67	Recurrent HPT	PTX with STX	Left	2	L1	Traction	Gradual	Yes	2726	6.88	Normal
Female, 13	MTC	TT + CND + LND	Left	1	Vagus^c^	Pressure	Undiscovered until V2	Yes	375	5.5	Normal
Female, 77	Retrosternal MNG	HT left side with STX	Left	1	Vagus^d^	Pressure	Undiscovered until V2	No	316	8.5	Normal
Male, 74^e^	Retrosternal MNG	HT left side with STX	Left	2	L2	Traction	Unknown	No	5335	9.25	Transient palsy

*CND* central neck dissection, *HPT* hyperparathyroidism, *HT* hemithyroidectomy, *LND* lateral neck dissection, *MNG* multinodular goiter, *MTC* medullary thyroid cancer, *PTC* papillary thyroid cancer, *STX* sternotomy, *TT* total thyroidectomy

^a^Type 1: segmental nerve lesion; type 2: global nerve lesion

^b^For definition: see text and Table 3

^c^Due to pressure on the vagus nerve proximally to the APS electrode, LOS was undetected by CIONM but later by INS

^d^Unrecognized torsion of the APS electrode leading to segmental pressure on vagus nerve; despite normal CIONM signals throughout the operation, intermittent vagus nerve stimulation proximally to the APS electrode did not evoke any signal at the end of dissection (V2)

^e^CIONM malfunction, surgery using INS

### CIONM events

At least one CIONM event could be registered on 61 (73 %) of 84 sides that were operated using CIONM. Of a total of 138 CIONM events, 137 were due to decreasing amplitudes, 17 combined with increasing latencies (combined events), and only one due to isolated latency increase. Among the 137 amplitude events, 38 (57 %) on 41 sides were due to amplitude reduction by more than 50 % or LOS, resulting in system alert.

Transsternal surgery (10 events for 13 NAR; 77 %) or large goiter (17 events for 27 NAR; 63 %) was not associated with significant higher CIONM event rates in the remaining patients in our cohort.

Ninety-one of the 138 events (66 %) were classified as technical artifacts, 13 events (9.4 %) as intrinsic (threatening), and 34 (24.6 %) as uncertain (potentially threatening). Intrinsic or uncertain events occurred significantly more frequently during dissection dorsal of the thyroid near the ligament of Berry (L4) when compared to the other dissection procedures (L1–3) (Table 3). Amplitudes below 25 % of the baseline/LOS were found in only 3/91 (3 %) of the artifacts but in 19/47 (40 %) of events classified as intrinsic or uncertain (*p* < .001) (Table 4). Artifacts were similarly frequent with tube (80/122; 66 %) and needle electrodes (12/16; 75 %; n. s.).

**Table 3:** CIONM events and dissection stage

	Lower pole mobilization (L1)^a^	Upper pole mobilization (L2)^a^	Inferior dissection (L3)	Dorsal dissection (L4)	*p*
Total number of CIONM events	14	40	18	66	–
Intrinsic or uncertain events	3 (21 %)	7 (18 %)	4 (22 %)	33 (50 %)	<.002
Amplitude below 25 % baseline or LOS	2 (14 %)	2 (5 %)	1 (6 %)	17 (26 %)	<.02

^a^In large goiters, the upper pole (L2) was mobilized first. *CIONM* continuous neuromonitoring, *LOS* loss of signal

**Table 4:** Effect of surgical management on CIONM events

CIONM event	Combined event	Decreased amplitude (%)	*n*	Actions (*n*) (%)	Time per action (s)^a^	Signal restitution (*n*)	Total action time (min)	RP (*n*)
Artifact *n* = 91	No *n* = 85	≥25	84	12 (14)	52 (30;75)	7	20	0
		<25	1	1 (100)	120	0	2	0
	Yes *n* = 6	>25	4	4 (100)	30 (30:68)	3	4	0
		<25	2	2 (100)	82	1	3	0
Uncertain event *n* = 34	No *n* = 30	>25	18	14 (78)	60 (30;105)	7	16	0
		<25	12	12 (100)	90 (45;135)	2	8	0
	Yes *n* = 4	>25	4	4 (100)	60 (28;112)	2	5	0
		<25	0	–	–	–	–	–
Intrinsic lesion *n* = 13	No *n* = 9	>25	5	4 (80)	120 (105;135)	2	8	0
		<25	4	4 (100)	180 (165;210)	2	13	1^b^
	Yes *n* = 4	>25	1	1 (100)	60	1	1	0
		<25	3	3 (100)	180 (105;180)	0	7	1^c^
Total			138	61 (44)	60 (30;120)	27	99	2

*CIONM* continuous vagal intraoperative neuromonitoring, *RP* recurrent laryngeal nerve palsy

^a^Median (interquartile range)

^b^Transient complete vocal fold palsy

^c^Permanent vocal fold palsy

In two patients, the APS electrode induced lesions of the vagus nerve: in the first patient by perineural bleeding followed by decreased amplitude using INS and in the second patient by an undetected torsion of the electrode resulting in LOS at V2 (Table 2). Both patients had normal vocal cord function by laryngoscopy 1 day after surgery.

### Loss of signal (LOS)

Losses of signal occurred in seven patients: segmental on four and global on three sides. In four patients, the LOS was detected by the CIONM system. In two patients, the LOS was related to a segmental lesion of the vagus nerve, in one patient due to torsion of the APS electrode, and in a second patient due to a hook pressing on the vagus nerve proximal to the APS electrode. These two lesions were not discovered by CIONM and not before V2 testing using INS proximal to the APS electrode. In the third patient, LOS occurred during surgery using INS after initial CIONM malfunction.

**Fig. 3:**
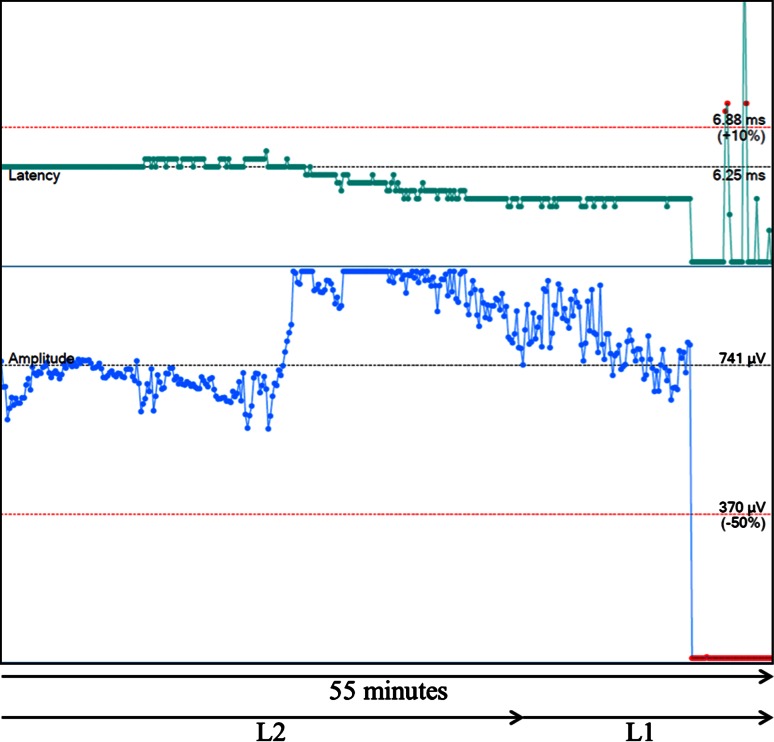
Acute segmental LOS (type 1) during mobilization of a mediastinal goiter on the right side in a 77-year-old female. Acute lesion during mobilization of the lower pole (L1), presumably due to traction. Outcome: Transient palsy right vocal fold

The spectrum of LOS type, cause, evolution, and outcome is given in Table 2. Two nerves in two patients recovered during surgery with normal postoperative function. Among the four patients in whom the LOS was detected by CIONM, two patients had acute LOS (Fig. 3), while LOS occurred gradually in the other two patients. Only patients with acute LOS were found to have postoperative vocal cord immobility.

### Corrective actions follow CIONM events

Of 138, 61 (44 %) CIONM events induced further corrective actions beyond a short relaxation of the thyroid gland or cervical compartment: tracheal compression and/or system check (*n* = 61), waiting (*n* = 49), the application of needle electrodes (*n* = 3), and intravenous administration of steroids (*n* = 3). Steroids were also given in the three patients with LOS that was not detected by CIONM. The procedures required a median of 60 s (interquartile range 30–120; Table 4); the short relaxation lasting a few seconds that was performed in all events was not registered. The signal was completely restored to prevent conditions in 27 of all 61 (44 %) events, but only in 16 of 49 (33 %) threatening or potentially threatening events. In the four patients with LOS and additional five patients with permanent amplitude below 50 %, surgery was continued under permanent alert despite LOS.

## Discussion

Vocal cord palsies are a common complication of endocrine neck surgery. Depending on diagnosis, surgical procedure, management of the RLN, and the experience and the skills of the surgeon, this complication occurs in up to 11 % of NAR [1]. The majority of RLN lesions (50–90 %) are reversible (transient), and in large volume centers, the frequency of permanent RLN palsies is below 1–2 % of NAR [1, 6, 14]. The high restitution rate of early vocal cord palsies reflects the underlying etiology of RLN lesions. The majority is caused by traction leading to potentially reversible neurapraxia or axonotmesis. It has been suggested that many of these lesions develop gradually over time, which—in theory—would facilitate their detection by CIONM before complete and possibly irreversible manifestation.

Even though the first published studies on CIONM reported very promising results [7, 9], the present series demonstrates a number of critical limitations of the current available CIONM devices.

1. EMG changes are frequent in the course of high-risk endocrine neck surgery. At least one system alert was triggered in about half of the dissected NAR, and EMG changes below 75 % of the baseline occurred in almost three quarters of all nerves. Two-thirds of events were due to technical artifacts. In recent years, translaryngeal needle electrodes have largely been replaced by endotracheal EMG electrodes fixed on intubation tubes [1, 2]. Given the latest improvements to the shape of currently available EMG endotracheal tubes, vertical or rotational dislocation under surgery no longer poses problems regarding stable EMG parameters. Endotracheal EMG tubes utilize mucosal contact electrodes that are not directly in contact with the intralaryngeal muscles cells. In addition to intralaryngeal tissue impedance, which might be affected by edema, the mechanical contact between tube and laryngeal wall has major impact on the amplitude of the recorded EMG signal. In large goiter with tracheal compression, thyroid surgery may induce weaker contact and thus lower amplitude by airway decompression. Even minor surgical maneuvers can produce significant reduction in the signal amplitude resulting in CIONM alert (Fig. 2).
2. A prerequisite of practicable and effective CIONM is the definition of reliable and dependable thresholds. Based on simple neurophysiological assumptions [7–9], a recent study using the NIM 3.0 device and APS system defined the combined event of reduced amplitude (below 50 % of the baseline) and increased latency (above 10 % of the baseline) as critical [9]. This prompted the manufacturer to preinstall these thresholds to trigger an acoustic alert. The reliability of combined amplitude and latency criteria has, however, never been proven. The latency in EMG of the RLN is composed of the nerve conduction velocity of the RLN of about 75 m/s [15] and the signal transfer across the neuromuscular junction. Depending on the distance between stimulation probe and signal detection electrode, the neuromuscular signal transfer will constitute more than 50 % of the latency (own unpublished data). Even if nerve conduction velocity was affected by imminent nerve lesions, the effect on global EMG latency would be consequently limited by the signal delay at the neuromuscular junction.
3. Some potentially reversible LOS may develop too fast to be prevented even by CIONM. In most of studies on conventional IONM, the frequency of vocal cord palsies was only marginally improved [1]. Even in high-risk surgery, the number of cases needed to treat (NNT) for prevention of a single RLN palsy is relatively high [5]. According to current international guidelines [2], the vagus nerve should be stimulated before starting any dissection near the RLN (V1) and at the latest possible time before finishing surgery (V2). This sequence is able to predict postoperative vocal fold function with positive and negative predictive values of 97–99 and 30–70 %, respectively [1, 14]. The number of stimulations during surgery is, however, limited. Consequently, INS will usually not detect a nerve lesion before it is complete. However, even CIONM with 10 stimulations per minute as in the NIM 3.0 device may not be frequent enough to prevent abrupt nerve lesions due to thermic damage or neurotmesis. In our series, two of the four LOS detected by CIONM occurred abruptly. Lesions that are manifest without delay do not permit the surgeon to take any protective action.
4. Segmental and global nerve lesions are indiscernible using stimulation of the vagus nerve as the localization of a voltage drop along the RLN requires INS. Whereas segmental LOS are caused by direct trauma (e.g., thermic, transection, clamping, but possibly also traction), the underlying pathophysiology is less certain in global LOS. Global LOS is supposed to result from traction during manipulation of the thyroid and the cervical airway segment. Damage is localized in the intralaryngeal segment of the nerve or in the neuromuscular junction and thus cannot be reached by conventional extralaryngeal IONM. It has been suggested that global lesions may develop gradually which makes them potentially preventable by CIONM. In our series, CIONM was, however, not able to detect more than four of the seven LOS while the false alerts due to artifactual EMG changes were frequent and time consuming. While the challenge of stable supramaximal stimulus application has been met by the latest systems (e.g., APS electrode, saxophone electrode, s-shaped electrode) [16–18], non-nerve damaging tissue manipulations such as traction of the trachea and the larynx cause EMG changes that cannot readily be distinguished from incipient neural damage.
5. A further important aspect of CIONM revealed in this series are neuronal lesions caused by the vagus electrode itself or lesions proximal to the vagus electrode. One LOS in our series was caused by the APS electrode itself, and the rate of temporary and partial lesion of the vagus nerve was 2 % due to the CIONM system. The problem of harm due to the APS system has only been reported once before, but should be recognized and integrated in the patient information [19].

The present study has a number of limitations. First, the restricted number of patients undergoing high-risk surgery limited the number of relevant clinical events (LOS and RLN palsy). Secondly, due to the non-randomized study design, no final conclusion regarding the potential benefit of CIONM can be drawn. Third, even though classification of events as intrinsic versus artifactual was based on several objective parameters, it ultimately rested on the subjective evaluation by the surgeon. Thus, we cannot exclude that some artifactual events were related to subclinical nerve damage and vice versa. Fourth, the protocol was not completely standardized (e.g., use of needle electrodes); however, this reflects clinical practice. Last, the results pertain to high-risk surgery and a specific device (APS) and can perhaps not be transferred to the systems by other manufacturers [16, 17].

In conclusion, CIONM using the APS system may be a useful tool in high-risk thyroid and parathyroid surgery in order to reduce the risk of RLN lesions. Even though the technology has matured, a major development effort is warranted to reduce EMG artifacts and improve the safety of CIONM systems. Randomized studies are needed in order to obtain reliable estimates of cost benefit.
